# Vitamin D deficiency in the *Apc^Pirc/+^* rat does not exacerbate colonic tumorigenesis, while low dietary calcium might be protective

**DOI:** 10.1242/dmm.032300

**Published:** 2018-03-01

**Authors:** Amy A. Irving, Elizabeth G. Duchow, Lori A. Plum, Hector F. DeLuca

**Affiliations:** Department of Biochemistry, University of Wisconsin-Madison, 433 Babcock Drive, Madison, WI 53706, USA

**Keywords:** Colon cancer, Vitamin D, Deficiency, Calcium, Rat model

## Abstract

Human studies have shown that individuals with colon cancer tend to have lower serum 25-hydroxy-vitamin D_3_ [25(OH)D_3_] levels compared with healthy controls, but whether this link is causative, a result of the disease or an indicator of another factor altogether has yet to be demonstrated. In humans, vitamin D, calcium and UV exposure are inextricably linked; therefore, understanding the individual and combined roles of each of these will require animal models specifically designed to address these questions. To begin to untangle this network, our group has employed the *Apc^Pirc/+^* rat, which contains a truncating mutation in the *Apc* gene, leading to the development of colonic tumors. Our group previously utilized this model to demonstrate that vitamin D supplementation above normal does not reduce colonic tumor burden and, in fact, increased tumor multiplicity in a dose-dependent manner. In the current study, we tested whether vitamin D deficiency plays a causative role in tumor development using two strains which differ in their susceptibility to intestinal tumorigenesis. In the colon, vitamin D deficiency did not increase the development of tumors in either strain, and was actually protective in one strain. Unexpectedly, low dietary calcium combined with vitamin D deficiency significantly suppressed tumor development in the small intestine and colon of both strains. The vast majority of tumors in the human intestine occur in the colon, and we find no evidence to support a direct role of vitamin D deficiency in increasing colonic tumorigenesis, and low calcium might protect against tumor development.

This article has an associated First Person interview with the first author of the paper.

## Abstract (precis)

**Summary:** Using rat models of familial and inflammation-induced colon cancer, this study demonstrates that vitamin D deficiency does not increase risk of developing disease, and that low dietary calcium might be protective.

## INTRODUCTION

It has been widely speculated that vitamin D can prevent the development of colonic adenomas. This suggestion has been based, to a large extent, on three general observations. First, colon cancer incidence is higher for individuals who live in locations further from the equator, which receive reduced amounts of sunlight, than for individuals who reside in locales near the equator (Cuomo et al., 2013). Second, several groups have demonstrated that patients with colon adenomas or cancers have lower serum 25-hydroxy-vitamin D_3_ [25(OH)D_3_] levels than those free from adenomas or cancers (Wu et al., 2007; Takahashi et al., 2010; Hong et al., 2012). Third, vitamin D compounds can inhibit the growth of cancer cells *in vitro* (Tangpricha et al., 2001; Kumagai et al., 2003; Bessler and Djaldetti, 2012; Wierzbicka et al., 2015). However, whether vitamin D deficiency plays a causative role in colon tumor development and, if so, whether this risk can be reversed with vitamin D supplementation of deficient individuals, has yet to be clearly demonstrated. Despite this, proponents continue to support an increase in the recommended daily allowance of vitamin D to reduce cancer risk (Vieth, 2011; Grant et al., 2016).

Determining the role of vitamin D deficiency in tumor development is a complex issue, as the vitamin D/calcium network is tightly regulated, and affecting one often affects the other. Further, vitamin D levels might simply be reflective of other factors, as body stores of this vitamin are affected by nutritional supplements, diet and exposure to UV. For these reasons, parsing out the individual contributions of each of these factors could prove nearly impossible in human populations. To vigorously test the contributing roles of each vitamin D, calcium and UV, animal models that closely mimic the disease in humans will be required.

The *Apc^Pirc/+^* rat, which contains a truncating mutation in the *Apc* gene, closely models the distribution, histopathology and morphology of both familial and sporadic colon cancer in humans (Amos-Landgraf et al., 2007; Irving et al., 2014). Using the *Apc^Pirc/+^* rat model, we have previously demonstrated that vitamin D supplementation above normal, with and without changes in serum calcium, does not reduce tumor incidence or growth. To the contrary, we found that addition of vitamin D_3_ to the diet of *Apc^Pirc/+^* rats increased tumor multiplicity in the colon in a dose-dependent manner (Irving et al., 2015). To further investigate this issue, we have explored whether vitamin D deficiency increases colon tumor development. We used two different modes to deplete rats of vitamin D to separate the effects of low calcium from those of low vitamin D. The first used low-calcium, vitamin D-devoid diets fed to weanling rats to create vitamin D deficiency. The second utilized rats conceived and born to mothers on a calcium-sufficient, vitamin D-deficient diet; these offspring were maintained on this diet for the duration of the study.

## RESULTS

### Experimental design

Two experimental approaches were taken to deplete animals of vitamin D. The first approach involved breeding parents devoid of vitamin D, but with sufficient calcium, to obtain pups always deficient in vitamin D but with normal levels of calcium. This method allowed us to isolate vitamin D deficiency without changes in serum calcium, and examine the extreme case where an individual is lacking in vitamin D for the entirety of its life. The second approach utilized rats born under normal dietary conditions where, at weaning, they were subjected to withholding of both vitamin D and calcium to deplete vitamin D systemically. This second method allowed us to obtain vitamin D deficiency in a timely manner by also removing calcium, and investigate the combined effect of vitamin D and calcium depletion, as calcium absorption is affected in individuals deficient in vitamin D.

**Fig. 1.**
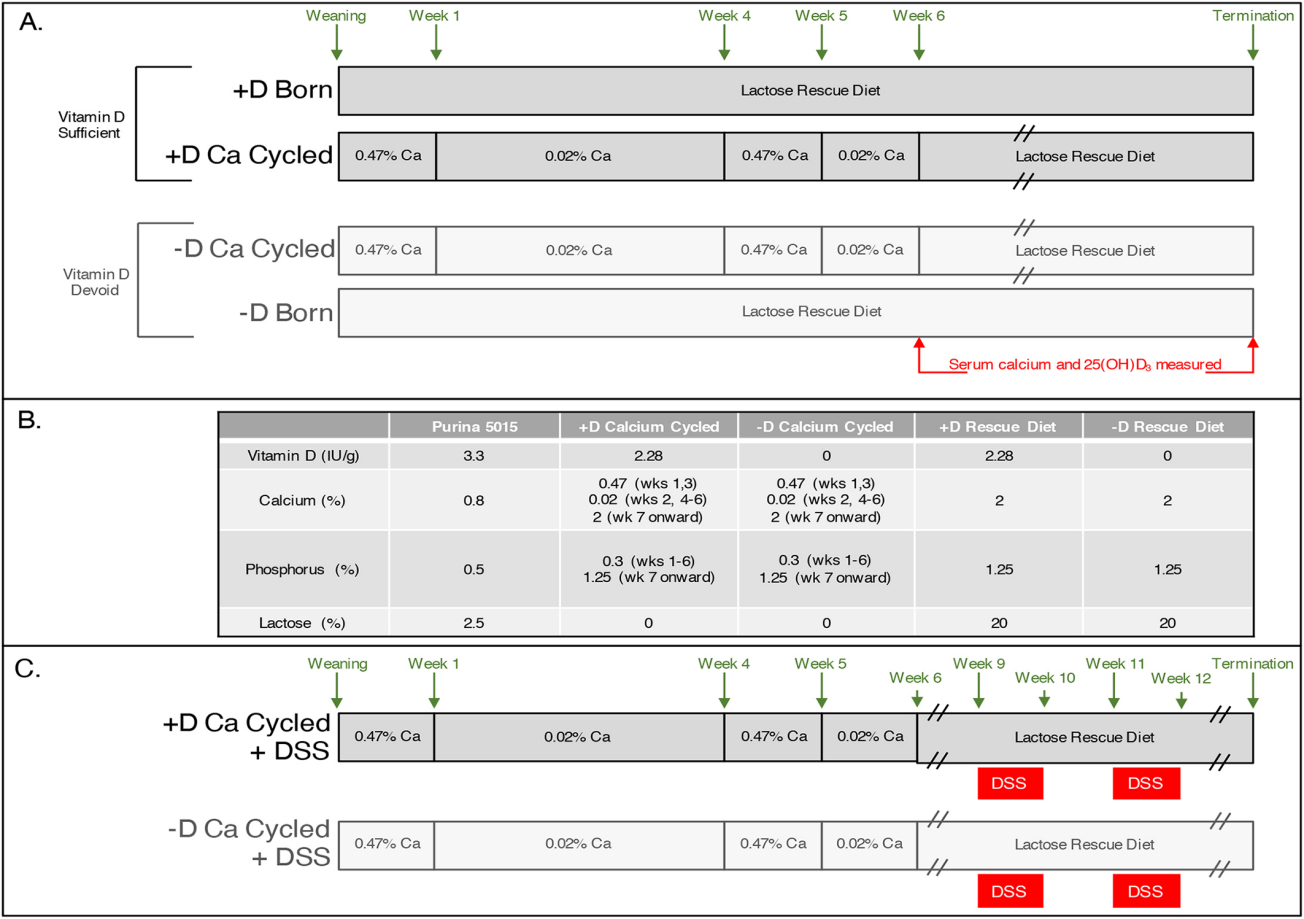
**Diagram of experimental diets and timeline.** (A) Rats born vitamin D sufficient on 5015 chow were randomized to one of three groups from weaning: lactose rescue diet containing normal amounts of vitamin D and calcium (+D Born); calcium-depleted, vitamin D normal diet for the first 6 weeks, followed by lactose rescue diet containing normal amounts of vitamin D and calcium (+D Ca Cycled); and calcium-depleted diet without vitamin D for the first 6 weeks followed by lactose rescue diet containing high amounts of calcium but lacking vitamin D (−D Ca Cycled). A fourth group contained animals born to vitamin D-deficient, calcium-sufficient parents, and weaned to a diet containing normal calcium but lacking vitamin D (−D Born). (B) Dietary vitamin D, calcium, phosphorus and lactose concentrations for the various diets used throughout the study. (C) For the experiment involving DSS, animals were assigned to a calcium-depleted diet with or without vitamin D for 6 weeks following weaning. Following cycling animals were placed on their respective lactose rescue diet (with or without vitamin D). Three weeks later, half of the animals on each of the diets were given 4% DSS in their drinking water for two week-long cycles, separated by a week on normal drinking water.

All rats, with the exception of those born to mothers on the vitamin D-deficient lactose rescue diet, were maintained on chow from conception to weaning; one group of control animals was maintained on chow until termination. At weaning, animals were assigned to one of two vitamin D-sufficient diets (vitamin D-sufficient lactose rescue diet or vitamin D-sufficient calcium-depleted diet) or depleted of vitamin D stores through the use of vitamin D-devoid, low-calcium diets (Fig. 1A,B). Following depletion, all animals were placed on to their respective lactose rescue diet with or without vitamin D_3_. Those animals born to mothers on vitamin D-deficient lactose rescue diet were maintained on that diet from conception until study termination. To induce inflammation-associated colonic tumorigenesis a separate set of (ACIxF344)F_1_ rats was calcium cycled in the presence or absence of vitamin D and then given two week-long treatments of 4% dextran sodium sulfate (DSS, FisherSci) separated by 1 week on regular drinking water (Fig. 1C).

### Blood calcium and 25(OH)D_3_ measurements

**Fig. 2.**
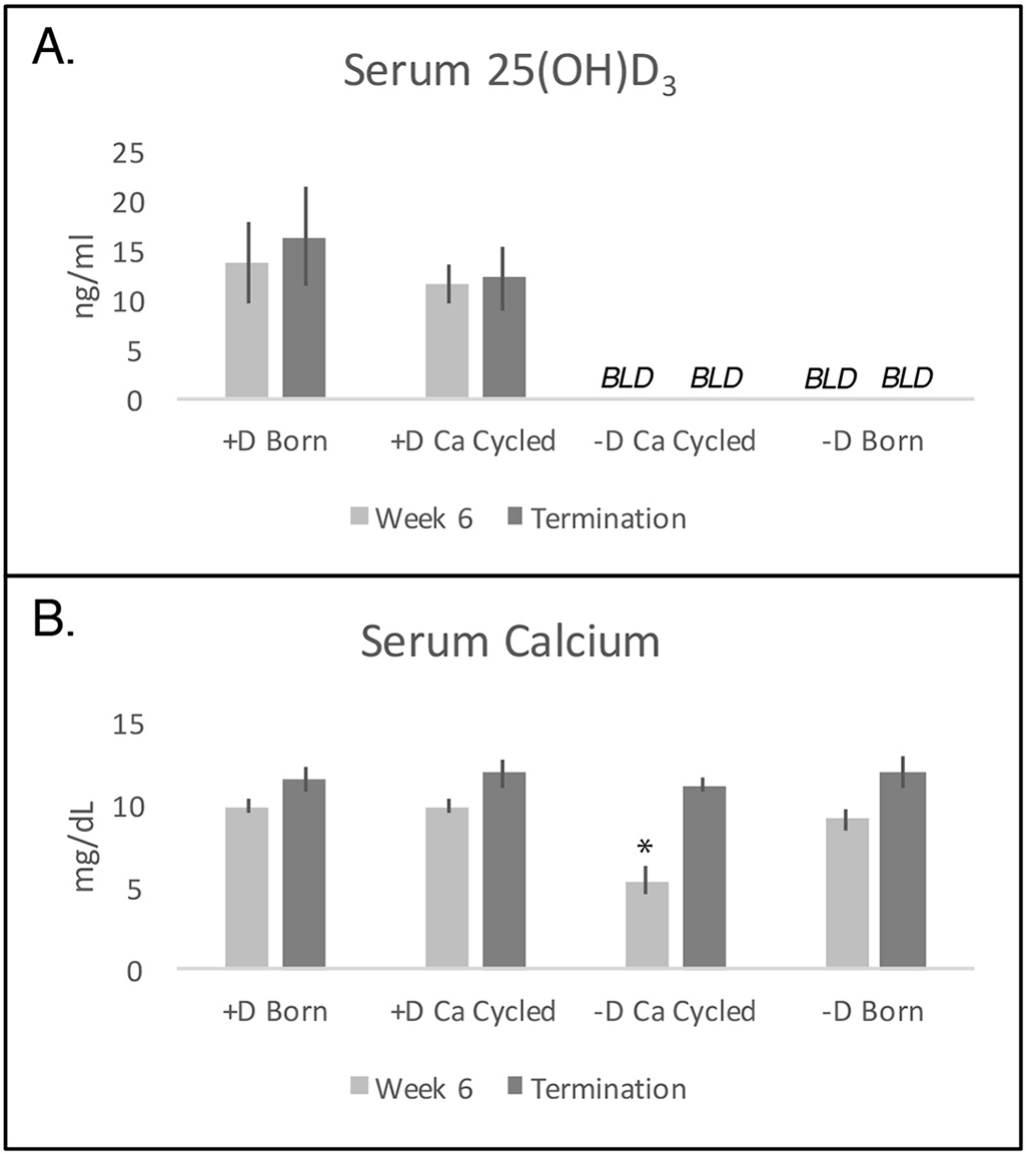
**Serum 25(OH)D_3_ levels were below the limit of detection for all rats considered vitamin D deficient and within normal range for vitamin D-sufficient rats.** Five rats per group per time point were analyzed for each biochemical assay. (A) Serum 25(OH)D_3_ levels did not differ between animals maintained on a vitamin D-sufficient lactose rescue diet and those given a vitamin D-sufficient, calcium-cycled diet (*P*=0.2). Serum 25(OH)D_3_ levels were below the limit of detection (BLD) for all animals given diets devoid of vitamin D (*P*<0.0001). (B) Serum calcium levels were significantly reduced only in vitamin D-deficient rats at the end of calcium cycling (*P<0.005 versus +D born, light-gray bars); these levels were restored to normal by termination (*P*=0.5 versus +D born, dark-gray bars). At the postcycling time point, calcium levels in animals born on the vitamin D-deficient lactose rescue diet (9.1±0.7) were slightly lower than those born on the vitamin D-sufficient diet (10.3±0.6, *P*<0.007); however, these levels were comparable at termination (12.0±1.1 versus 11.6±1.0, *P*=0.3). Data were analyzed using a two-tailed Wilcoxon test, and are expressed as mean±s.d.

The 6-week period of alternating dietary calcium (either 0.47% or 0.02%) and no dietary vitamin D successfully depleted blood levels of 25(OH)D_3_ (Fig. 2A, light-gray bars) below the limit of detection, which is also reflected in the low serum calcium levels (Fig. 2B, light-gray bars). Within 3 weeks on lactose rescue diet (high in calcium, lactose and phosphorus), blood calcium levels in these animals were restored to those of control animals while maintaining serum 25(OH)D_3_ levels below the limit of detection (data not shown). This remained true at study termination (Fig. 2A,B, dark-gray bars).

### Body weight measurements

**Fig. 3.**
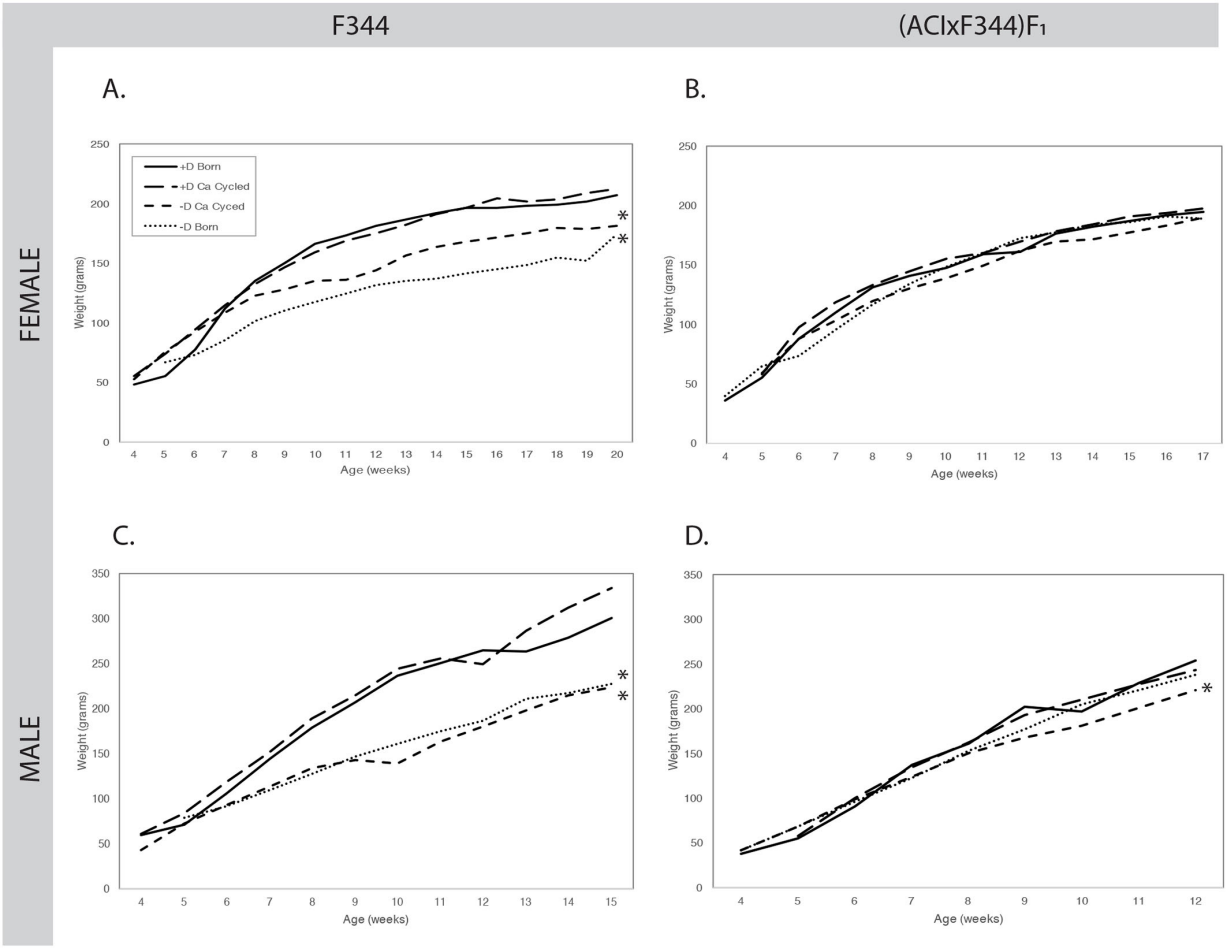
**Body weight gain is generally reduced in vitamin D-deficient rats.** (A-D) For vitamin D-sufficient groups, calcium cycling did not affect body weight in either sex or strain. F344-*Apc^Pirc/+^* females (A) and males (C) reared on either vitamin D-deficient diet showed significantly reduced weight gain during the duration of the experiment (*P<0.003). By contrast, in the (ACIxF344)F_1_*-Apc^Pirc/+^* rat, while body weight in females was unaffected (B), vitamin D depletion reduced body weight gain only in males in the calcium-cycled group (D, *P=0.003). Data were analyzed using a two-tailed Wilcoxon test. The number of rats in each group were as follows (female, male): F344 +D Born (9, 9), +D Ca Cycled (6, 5), −D Ca Cycled (5, 10), −D Born (6, 5); (ACIxF344)F_1_ +D Born (20, 20), +D Ca Cycled (10, 6), −D Ca Cycled (11, 17), −D Born (5, 4).

Elimination of vitamin D from the diet and depletion of body stores of calcium and vitamin D, as measured by circulating blood levels of 25(OH)D_3_, significantly reduced body weight gain in female (*P*<0.001, Fig. 3A) and male (*P*<0.003, Fig. 3C) F344*-Apc^Pirc/+^* rats. By contrast, in the (ACIxF344)F_1_*-Apc^Pirc/+^* rat, vitamin D depletion only reduced body weight gain in males in the calcium-cycled group (*P*=0.003, Fig. 3D), but the suppression in weight gain compared to vitamin D-sufficient born rats was less (13%) compared to that in F344*-Apc^Pirc/+^* male rats (26%); females were unaffected (Fig. 3B). Diet low in calcium but with normal vitamin D levels did not negatively impact body weight in either strain.

### Tumor multiplicity and sizing data

**Fig. 4.**
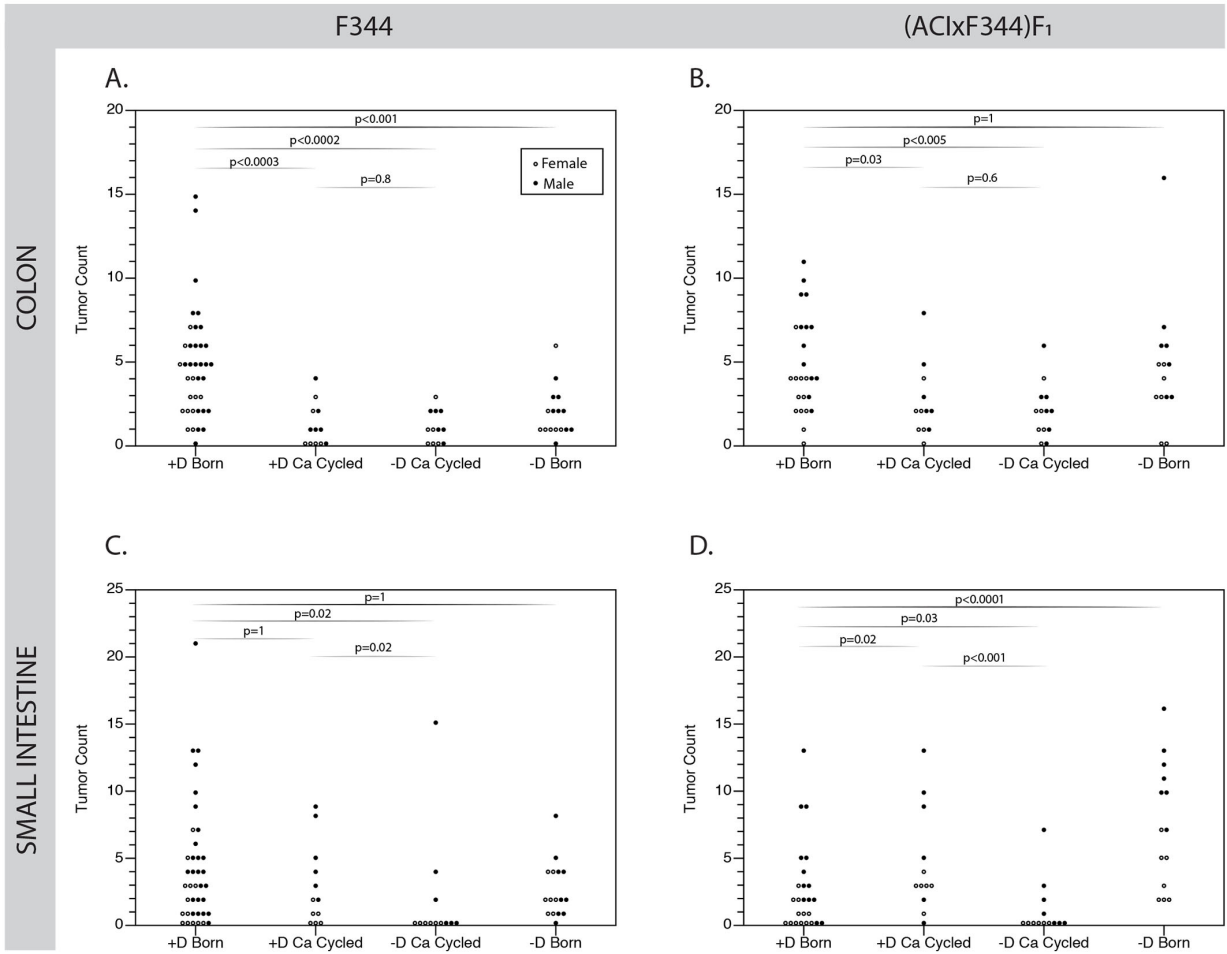
**Vitamin D deficiency did not increase colonic tumorigenesis in either strain.** (A) In the colon of the F344-*Apc^Pirc/+^* rat, tumor multiplicities were reduced in animals given a diet devoid in vitamin D (*P*<0.001); reduced dietary calcium alone also significantly reduced tumor number (*P*<0.0003). (B) In the colon of the (ACIxF344)F_1_*-Apc^Pirc/+^* rat, there was no effect of vitamin D depletion alone on tumor multiplicity; however, reduction of dietary calcium alone (*P*=0.03) or in combination with vitamin D deficiency (*P*<0.005) reduced colonic tumor number. (C) For the small intestine, tumor count in both strains was significantly reduced when low dietary calcium was combined with vitamin D deficiency (*P*<0.03). By contrast, in the small intestine of the (ACIxF344)F_1_*-Apc^Pirc/+^* rat, tumor multiplicity was increased in rats given a diet absent in vitamin D but normal in calcium (*P*=0.0001) or vice versa (*P*=0.02). Each individual point represents data from a single animal. Open circles represent females, while closed circles represent males; all data were blocked by sex and then analyzed jointly. Data were analyzed using a two-tailed Wilcoxon test. All *P*-values shown have been Bonferroni corrected for multiple testing against a single control group.

No statistical difference in tumor number for either the colon or the small intestine was found between rats maintained on 5015 chow and those weaned to vitamin D-sufficient lactose rescue diet for either strain (all *P*>0.13); therefore, these data were combined to yield one single vitamin D sufficient control group for each strain and sex for all other comparisons. Compared with vitamin D-sufficient controls, rats born on a vitamin D-deficient diet with normal serum calcium levels had either a reduction in the number of colonic tumors (F344, *P*<0.001, Fig. 4A) or no change (F_1_, *P*=1, Fig. 4B). Surprisingly, in both strains a low-calcium diet reduced colonic tumor multiplicity, regardless of whether vitamin D was present or absent (all *P*<0.03, Fig. 4A,B).

Colonic tumors were measured in (ACIxF344)F_1_*-Apc^Pirc/+^* male rats born on either vitamin D-sufficient lactose diet (*n*=6) or vitamin D-deficient lactose diet (*n*=7) to determine whether vitamin D plays a role in tumor growth after initiation. No difference in average tumor size was found between those animals with sufficient vitamin D levels (8.2±7.9 mm^2^, *n*=38) and those deficient in vitamin D (7.1±6.3 mm^2^, *n*=39, *P*=0.66).

In the small intestine, tumor multiplicity was not affected in F344-*Apc^Pirc/+^* rats born on a vitamin D-deficient, calcium-normalized diet (Fig. 4C); only the combination of low dietary calcium and vitamin D deficiency lowered the number of tumors present (*P*=0.02). By contrast, (ACIxF344)F_1_*-Apc^Pirc/+^* rats given a vitamin D-deficient, calcium-normalized diet showed a significant increase in the number of tumors in the small intestine for both male and female rats (*P*<0.0001, Fig. 4D). Interestingly, lowering dietary calcium alone for a period of 6 weeks also increased the number of tumors in the small intestine in the (ACIxF344)F_1_*-Apc^Pirc/+^* rats (*P*=0.009). However, when vitamin D and calcium were both depleted, tumor number was decreased in both strains in both the small intestine and the colon (all *P*<0.03).

**Fig. 5.**
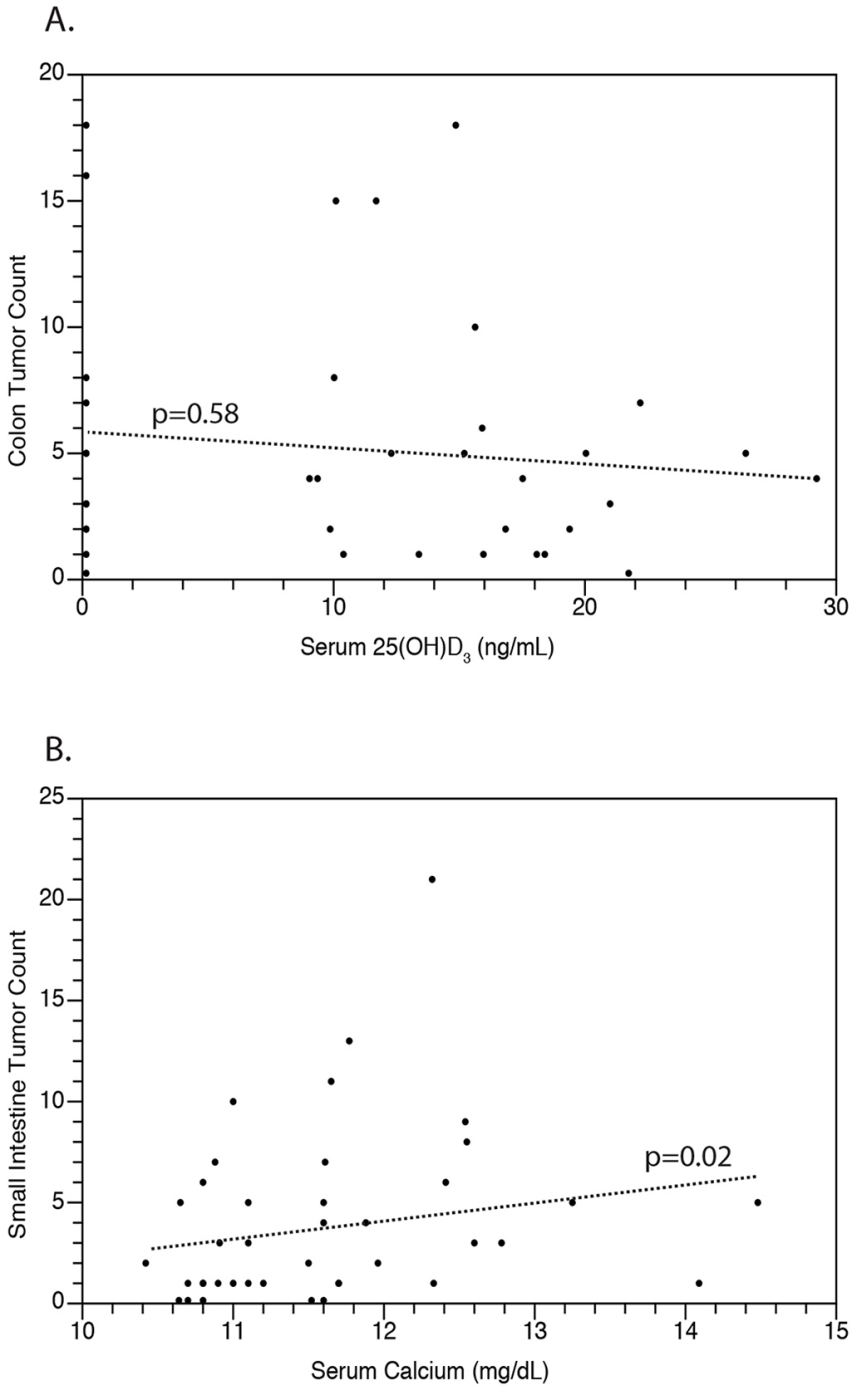
**Colonic tumor count does not correlate with serum 25(OH)D_3_ or calcium levels.** (A) No correlation was found between serum 25(OH)D_3_ levels and tumors of either the small intestine (data not shown) or colon (*P*=0.58). (B) A significant positive association was uncovered between serum calcium levels and tumors of the small intestine (*P*=0.02) but not the colon (data not shown). Data were analyzed using Kendall's test for correlation. Each individual point represents data from a single animal. At least five rats per dietary group are represented.

Data were further analyzed to test for a correlation between colonic tumor multiplicity and serum 25(OH)D_3_ concentration; no such correlation was found (*P*=0.58, Fig. 5A). By contrast, a significant positive correlation between tumor count in the small intestine and serum calcium concentration was detected (*P*=0.02, Fig. 5B).

**Fig. 6.**
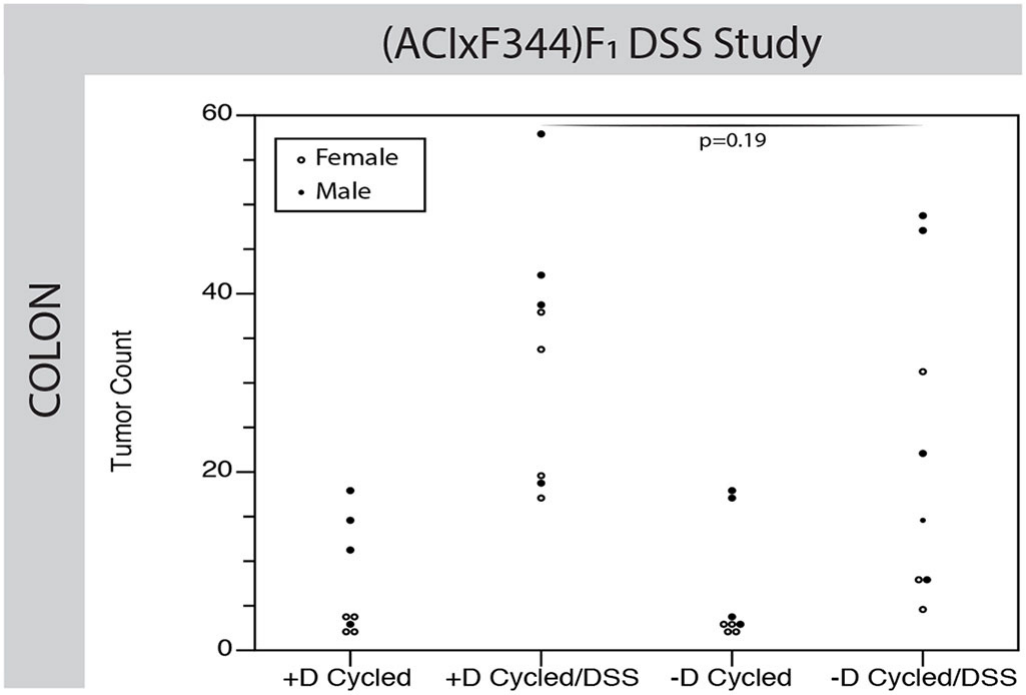
**Vitamin D deficiency did not affect tumor number in the DSS model of tumorigenesis.** In the set of (ACIxF344)F_1_-*Apc^Pirc/+^* rats utilized to test the effect of vitamin D on colon cancer induced by inflammation, no difference in response to DSS was found between vitamin D-sufficient and -deficient animals. DSS increased tumor multiplicity for both vitamin D-sufficient and -deficient animals to a similar degree (*P*<0.005). No difference in colonic tumor multiplicity was found between vitamin D-deficient and -sufficient animals with (*P*=0.19) or without (*P*=0.7) DSS treatment. Each individual point represents data from a single animal. Open circles represent females, while closed circles represent males; all data were blocked by sex and then analyzed jointly. Data were analyzed using a two-tailed Wilcoxon test.

To test whether vitamin D status affects tumor development in a model of colon cancer induced by inflammation, a separate cohort of (ACIxF344)F_1_*-Apc^Pirc/+^* rats was calcium cycled in either the presence or absence of vitamin D, and then given DSS to induce colonic inflammation. Again, we saw no difference in colonic tumor number between vitamin D-sufficient and -deficient rats not given DSS (*P*=0.75, Fig. 6). Each of the groups treated with DSS, regardless of vitamin D status, showed a statistically significant increase in colonic tumorigenesis (*P*<0.005) compared with non-DSS-treated controls. There was no difference in the number of colon tumors between vitamin D-sufficient rats treated with DSS and vitamin D-deficient rats given DSS (*P*=0.19), demonstrating that vitamin D status did not affect the DSS response.

### Tumor pathology

A minimum of 20 individual tumor samples from four rats per group were selected for sectioning. Sixty 5 µm sections (300 µm) were made from each tumor, with every 10th section (50 µm) stained by Hematoxylin and Eosin for evaluation of pathology. At least half of the tumors for any given group yielded sections allowing pathology review. Tumors were graded using a five-point scale of malignancy: 1, adenoma; 3, intramucosal carcinoma; 5, early carcinoma; intermediate grades 2 and 4 were assigned when a lesion exhibited some, but not all, criteria for the next grade. Based on this scoring system, the vast majority of tumors were classified as adenomas in each of the groups: +D calcium cycled 1.8±0.9, +D calcium cycled+DSS 1.9±0.8, −D calcium cycled 1.8±0.9, −D calcium cycled+DSS 2.2±0.9. No significant differences in malignancy were detected between any of the groups (all *P*>0.23).

### *Mki67* gene expression

**Fig. 7.**
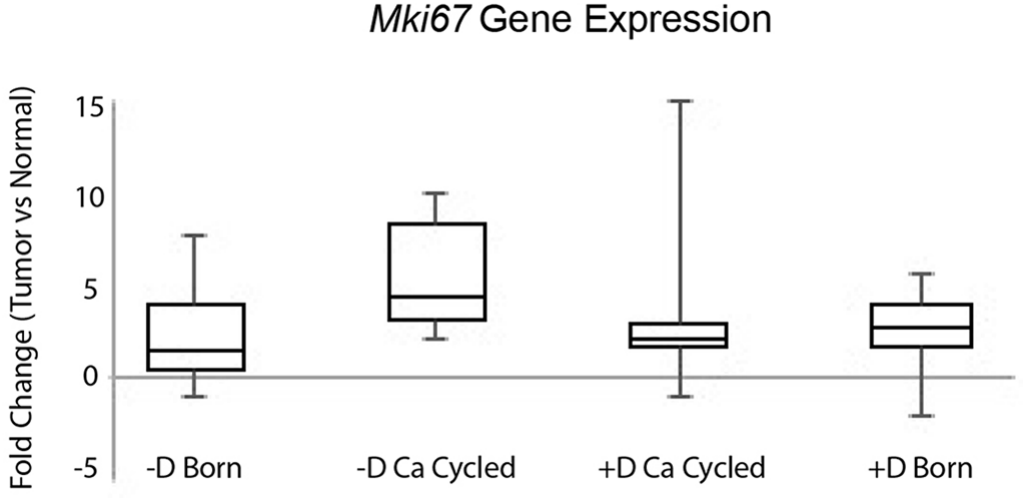
**Vitamin D deficiency did not alter cell proliferation rates in tumors, as measured by *Mki67* gene expression.** *Mki67* was measured in matched pairs of normal colonic tissue and colonic tumors from 7-10 rats per treatment group. Expression is represented as fold change in tumor expression versus normal tissue expression; all values have been normalized against the reference gene *Gapdh*. In general, tumor expression was higher than normal colonic tissue expression. However, no significant difference in tumor *Mki67* gene expression was detected between any of the groups, indicating that vitamin D deficiency does not influence tumor cell proliferation rates *in vivo* (all *P*>0.27). The number of tumor/normal pair-matched samples analyzed per group were as follows: −D Born (7), −D Ca Cycled (7), +D Ca Cycled (7), +D Born (10).

To examine cell proliferation rates, *Mki67* gene expression was assessed by quantitative PCR from matched pairs of normal colonic tissue and colonic tumors from at least seven rats per dietary group. For all groups, tumors generally showed higher expression of *Mki67* compared with their matched normal tissue, indicating a higher rate of cell proliferation in tumors (Fig. 7). However, no significant difference in tumor cell proliferation was detected between any of the groups to indicate that a particular diet might result in an increase in cell proliferation (all *P*>0.27). In normal colonic tissue, *Mki67* expression, and thus cell proliferation, was lower for animals that had been made vitamin D deficient by calcium cycling, compared with rats born vitamin D sufficient (*P*=0.006); no other differences in proliferation of normal tissue were noted between any of the other groups.

## DISCUSSION

The results presented here add valuable information to our understanding of the functional outcome of vitamin D deficiency on intestinal cancer. Two different modes were employed to generate vitamin D deficiency in two *Apc^Pirc/+^* rat models of familial adenomagenesis, as well as extending this model to address inflammatory tumorigenesis. In any case, a lack of dietary vitamin D, resulting in undetectable levels of serum 25(OH)D_3_, did not result in an increase in colonic tumor multiplicity. In fact, the opposite was seen in the F344 rat, where vitamin D depletion significantly reduced colonic tumorigenesis. Deficiency of vitamin D also did not affect the growth rate of tumors once established, nor the degree of malignancy. Furthermore, no association between serum 25(OH)D_3_ levels and colon tumor number was detected.

By contrast, in the small intestine, vitamin D deficiency did increase the number of tumors. Concordantly, our group has previously reported a statistically significant dose-dependent reduction in the number of tumors of the small intestine with 25(OH)D_3_ supplementation, while at the same time seeing the opposite trend in the colon (Irving et al., 2015). It is important to recognize that the majority of tumors in the human intestine form in the colon (Yi Pan et al., 2011), and this difference in anatomical site within the intestine might have been historically underemphasized when employing mouse models in which the tumor burden predominantly occurs in the small intestine (Irving et al., 2014). This result does not overshadow our finding that is most translatable to the human case: vitamin D deficiency did not increase tumor development or growth in the colon.

Unexpectedly, low dietary calcium alone significantly reduced tumor number in the colon in both rat models. In the small intestine, lower serum calcium levels were also associated with a reduction in tumor number. Epidemiological evidence has previously pointed to a protective effect of dietary calcium (Bailie et al., 2017; Zhang et al., 2016). However, results from clinical trials have yielded mixed results for supplemental calcium on reducing primary or recurrent cancer (Carroll et al., 2010; Pommergaard et al., 2016) or modulating biomarkers of inflammation or oxidative stress in colorectal mucosa (Danese and Mantovani, 2010; Fedirko et al., 2011; Hopkins et al., 2011; Ahearn et al., 2012; Yang et al., 2015). These results warrant further investigation of the individual and combined effects of calcium and vitamin D on the normal intestine and their tumors.

While a great deal of attention has been paid to the association between serum 25(OH)D_3_ levels and disease, few studies have been conducted that interrogate the effect of vitamin D deficiency on intestinal tumor development. Those that do exist generally rely on the use of knockout models for genes encoding enzymes and receptors in the vitamin D pathway. Given the complexity of the vitamin D pathway, involving many enzymes, organs and intermediate metabolites, these models might be inappropriate to test whether vitamin D deficiency affects colon cancer risk, and could actually confound interpretation. For example, Liu and colleagues found that animals lacking the *Cyp27b1* gene, which encodes the enzyme responsible for conversion of 25(OH)D_3_ to the active metabolite 1,25(OH)_2_D_3_, show colonic inflammation and signs of cellular senescence (Liu et al., 2016). However, *Cyp27b1* null mice have elevated 25(OH)D_3_ blood levels (Wang et al., 2012), and we have previously shown that elevated 25(OH)D_3_ exacerbates colonic tumorigenesis (Irving et al., 2015). The intricacy of this tightly regulated network requires analysis of the system as a whole, and in a way that accurately mimics vitamin D deficiency in humans. Here, we show that elimination of vitamin D from the diet and environment had no negative impact on subsequent colon tumor development.

While the debate of whether vitamin D status is causally linked to colon cancer risk is ongoing, other hypotheses warrant simultaneous attention. One such hypothesis harkens back to the original observation between geographic latitude and colon cancer risk – UV light exposure. Recently, Rebel and colleagues found that UV exposure of Fabpl*Cre*; *Apc*^15lox/+^ mice reduced overall tumor burden, which they at least partially attribute to vitamin D production (Rebel et al., 2015). Vitamin D production is one significant benefit and marker of sunlight exposure, but it is likely that other factors produced by UV light also support human health (van der Rhee et al., 2013). It is still unknown whether vitamin D status in human studies of disease association is simply a reflection of UV exposure (Yang and Toriola, 2017). This will likely be very difficult to disentangle using human studies alone and will require the use of appropriate animal models to vigorously test. The individual and combined effects of vitamin D, calcium, and UV radiation require further investigation in animal models specifically designed to address each component in order to elucidate a complete and accurate picture of their effects on colon cancer risk.

## MATERIALS AND METHODS

### Animal breeding and maintenance

All procedures were approved by the Research Animal Resources Committee of the College of Agricultural and Life Sciences at the University of Wisconsin-Madison. Rats (*Rattus norvegicus*) were maintained in high-density ventilated caging (typically two to three rats per cage) with corncob bedding in the Department of Biochemistry vivarium, with a 12 h:12 h light:dark cycle, and free access to food (Purina 5015 chow, unless noted otherwise) and UV-treated RO water. During the period of calcium cycling, rats were single housed in wire-bottom cages with a loft for resting, to reduce cophrophagy and thus reuptake of vitamin D and calcium from fecal material; free access to food and water was maintained during this period. Fluorescent bulbs in rooms housing rats were covered by filters which eliminate the wavelengths responsible for the majority of vitamin D conversion in skin. The *Apc^Pirc/+^* rat, a model of both familial and sporadic intestinal tumorigenesis, contains a truncating mutation in the *Apc* gene. This results in the development of multiple intestinal adenomas, with a larger proportion forming in the colon rather than the small intestine, similar to the distribution seen in humans, and different from many available mouse models. F344-*Apc^Pirc/+^* rats were maintained through breeding with F344/NHsd rats obtained from Envigo (Dublin, VA). Coisogenic F344-*Apc^Pirc/+^* rats have approximately a 1:1 ratio between tumors in the small intestine and in the colon; to shift that ratio to obtain a greater number of tumors in the colon, F_1_ rats between F344 and ACI were generated (Washington et al., 2013). F_1_ generation rats were created between breeding pairs of F344-*Apc^Pirc/+^* rats with ACI/SegHsd rats obtained from Envigo (Indianapolis, IN), resulting in (ACIxF344)F_1_-*Apc^Pirc/+^* progeny.

### Diets

Diets were formulated in our laboratory and stored for up to 3 months at 4°C. Animals were given fresh diet three times each week *ad libitum*. Lactose rescue diets contained 2% calcium plus 20% lactose to aid in calcium absorption in the absence of vitamin D; deficient and sufficient diets were identical except for vitamin D content. Diets used to deplete rats of their vitamin D stores contained no vitamin D and either 0.47% or 0.02% calcium, depending on the stage of depletion (Fig. 1A); corresponding sufficient diets were identical but also contained vitamin D (2280 IU cholecalciferol per 1 kg diet). Vitamin D-deficient diets are prepared in a room which is never exposed to vitamin D compounds or analogs and stored in a dedicated cooler; vitamin D-sufficient diets are prepared in a separate room and stored in a separate cooler.

### Experimental design

All rats, with the exception of those born to mothers on the vitamin D-deficient lactose rescue diet, were maintained on 5015 chow (Purina) from conception to weaning; one group of control animals was maintained on 5015 chow until termination. At weaning, animals were assigned to one of two vitamin D-sufficient diets (vitamin D-sufficient lactose rescue diet or vitamin D-sufficient, calcium-depleted diet) or depleted of vitamin D stores through the use of vitamin D-devoid, low-calcium diets (Fig. 1A,B). Animals within a single litter were randomized to multiple groups when possible to reduce litter- or parent-specific confounders. Following depletion, all animals were placed on their respective lactose rescue diet with or without vitamin D_3_. Those animals born to mothers on the vitamin D-deficient lactose rescue diet were maintained on that diet from conception until study termination. F344 rats were euthanized at either 120 (male) or 150 (female) days of age; (ACIxF344)F_1_ rats were euthanized at either 90 (male) or 120 (female) days of age. Euthanization time points were determined empirically for each strain and sex to maximize the number of tumors per animal, while avoiding morbidity from the disease. To induce inflammation-associated colonic tumorigenesis, a separate set of (ACIxF344)F_1_ rats was calcium cycled in the presence or absence of vitamin D, and then given two rounds (each lasting 1 week) of 4% (wt/vol) DSS (FisherSci) separated by 1 week on regular drinking water (Fig. 1C). All rats in the DSS experiment were terminated at 140 days of age.

### Sample collection, terminal tumor counts and sizing

At sacrifice, the small intestine and colon were removed, washed with PBS and laid flat. The entire intestine was fixed with 10% neutral buffered formalin for 48 h and then transferred to 70% ethanol, where they were stored until further use. Total tumor counts for the small intestine and colon were obtained on a dissecting microscope at 10× magnification by a blinded observer. Tumor dimensions were measured to the nearest 0.1 mm using an eyepiece reticule at 10× magnification. Two measurements were taken in the plane of the epithelium for each tumor: the longest tumor dimension was measured first (A), followed by a second measurement perpendicular to the first (B). To determine an area for each tumor, the equation for area of an ellipse was used: π(A/2)(B/2).

### Tumor pathology

After tumor counts were obtained from fixed sections, samples were collected for histology and review by a pathologist familiar with tumors in this model. At least 20 individual tumor samples from four rats per group from the DSS study were selected for sectioning. Tumors were bisected vertically through the stalk, embedded and cut into 5 µm sections, for a total of 60 sections (300 µm). Every 10th section (50 µm) was stained by Hematoxylin and Eosin for evaluation of pathology. Tumors were graded using a five-point scale of malignancy: 1, adenoma; 3, intramucosal carcinoma; 5, early carcinoma; intermediate grades 2 and 4 were assigned when a lesion exhibited some but not all criteria for the next grade.

### Biochemical assays

Blood was obtained at week 6 from the lateral ocular orbit and at termination by cardiac puncture to assess calcium and vitamin D concentrations. A minimum of five animals per diet per time point were evaluated for each serum calcium and 25(OH)D_3_. Serum calcium was measured by atomic absorption spectrometry on the Perkin Elmer 900H instrument. Serum 25(OH)D_3_ concentrations were measured by high-performance liquid chromatography (HPLC), as described previously (Irving et al., 2015). The HPLC method has been confirmed to separate 25(OH)D_3_, 24,25-dihydroxy-vitamin D_3_ [24,25-(OH)_2_D_3_] and 1,25-dihydroxy-vitamin D_3_ [1,25-(OH)_2_D_3_]. Extraction recoveries were checked using tritiated 25(OH)D_3_ each time samples were processed and averaged 95%. Two 25(OH)D_3_ standards were analyzed for every 10-14 experimental samples; this was flanked by one background check each at the beginning and the end of the run. The extracted and filtered samples (total volume of 250 ml) were loaded on a Symmetry C18 column (5 μm, 3.9 mm×150 mm; Waters) at 30°C, and UV absorbance was measured at 265 nm at a flow rate of 1 ml/min under a reversed phase isocratic method. Blood concentrations of 25(OH)D_3_ were calculated on the basis of the extraction/filtration/resuspension recoveries and a standard curve spanning 0.5 to 50 ng, with a lower limit of quantification of 5 ng/ml serum and a lower limit of detection of 3 ng/ml serum.

### Expression analysis

Samples of colonic tumors and normal colonic tissue were collected at dissection and stored in RLT Plus Buffer (Qiagen, Valencia, CA) at −80°C. Normal tissue was collected outside a minimum 2-mm barrier surrounding any tumor. RNA was isolated using an Allprep DNA/RNA Mini Kit (Qiagen) following the manufacturer's protocol. cDNA was generated using a High Capacity cDNA Reverse Transcription Kit (Applied Biosystems, Foster City, CA). Taqman hydrolysis probes from Applied Biosystems for *Mki67* (FAM, Rn01451446_m1) and *Gapdh* (VIC Primer Limited, 4352338E) were duplexed in a single well for each sample, and each sample was run in duplicate on a StepOne Plus Real Time PCR System (Applied Biosystems). *Gapdh* was used as a reference gene, and each tumor sample was pair matched to its own normal tissue for analysis. Seven to ten rats per dietary group were examined. Fold change values represent the expression in a tumor sample compared with its own normal tissue sample, each normalized to the reference gene *Gapdh*.

### Statistical methods

In the *Apc^Pirc/+^* rat, there are significant differences between the number of tumors in the small intestine and in the colon (Amos-Landgraf et al., 2007); therefore, these two areas have been analyzed separately. The means of tumor multiplicity data are not normally distributed and therefore require the use of nonparametric statistics; thus a two-sided Wilcoxon rank sum test was employed. Tumor multiplicities in the *Apc^Pirc/+^* rat show significant sex differences (Amos-Landgraf et al., 2007); therefore, multiplicity data were blocked based on Lehman's extension to the Wilcoxon rank sum test and jointly tested for the effects of the test conditions (Lehmann, 1998). A minimum of 12 rats per diet per strain were analyzed for the vitamin D deficiency studies. This group size gives 90% power at significance level of 0.05 (two-tailed) to detect either a decrease by 50% or a doubling in colon tumor number compared to control. All statistics were performed using the freely available software MSTAT (Drinkwater, 2017). A Bonferroni-corrected *P*-value has been used in instances with multiple comparisons to the same control. No difference between sexes or strains was observed for the serum biochemical assays; therefore, these data have been combined and analyzed using a two-sided Wilcoxon rank sum test. To test for an association between tumor multiplicity and biochemical assay measures, a two-sided Kendall's rank correlation test was used. *P*≤0.05 was considered significant for all tests.

## Supplementary Material

First Person interview

## References

[DMM032300C1] Ahearn T. U., Shaukat A., Flanders W. D., Rutherford R. E. and Bostick R. M. (2012). A randomized clinical trial of the effects of supplemental calcium and vitamin D3 on the APC/B-catenin pathway in the normal mucosa of colorectal adenoma patients. *Cancer Prev. Res.* 5, 1247-1256. 10.1158/1940-6207.CAPR-12-0292 PMC3466388 22964475

[DMM032300C2] Amos-Landgraf J. M., Kwong L. N., Kendziorski C. M., Reichelderfer M., Torrealba J., Weichert J., Haag J. D., Chen K.-S., Waller J. L., Gould M. N. et al. (2007). A target-selected Apc-mutant rat kindred enhances the modeling of familial human colon cancer. *Proc. Natl. Acad. Sci. USA* 104, 4036-4041. 10.1073/pnas.0611690104 17360473 PMC1805486

[DMM032300C3] Bailie L., Loughrey M. B. and Coleman H. G. (2017). Lifestyle risk factors for serrated colorectal polyps: a systematic review and meta-analysis. *Gastroenterology* 152, 92-104. 10.1053/j.gastro.2016.09.003 27639804

[DMM032300C4] Bessler H. and Djaldetti M. (2012). 1a,25-dihydroxyvitamin D3 modulates the interaction between immune and colon cancer cells. *Biomed. Pharmacother.* 66, 428-432. 22795808 10.1016/j.biopha.2012.06.005

[DMM032300C5] Carroll C., Cooper K., Papaioannou D., Hind D., Pilgrim H. and Tappenden P. (2010). Supplemental calcium in the chemoprevention of colorectal cancer: a systematic review and meta-analysis. *Clin. Ther.* 32, 789-803. 10.1016/j.clinthera.2010.04.024 20685491

[DMM032300C6] Cuomo R. E., Mohr S. B., Gorham E. D. and Garland C. F. (2013). What is the relationship between ultraviolet B and global incidence rates of colorectal cancer? *Dermatoendocrinology* 5, 181-185. 10.4161/derm.23773 PMC3897587 24494052

[DMM032300C7] Danese S. and Mantovani A. (2010). Inflammatory bowel disease and intestinal cancer: a paradigm of the Yin-Yang interplay between inflammation and cancer. *Oncogene* 29, 3313-3323. 10.1038/onc.2010.109 20400974

[DMM032300C8] Drinkwater N. (2017). *MSTAT Statistical Software* Available at: https://mcardle.wisc.edu/mstat/download/index.html (Accessed: 10 March 2017).

[DMM032300C9] Fedirko V., Bostick R. M., Long Q., Flanders W. D., Marjorie L., Sidelnikov E., Daniel C. R. and Rutherford R. E. (2011). Effects of supplemental vitamin D and calcium on oxidative DNA damage marker in normal colorectal mucosa: a randomized clinical trial. *Cancer Epidemiol. Biomarkers Prev.* 19, 280-291. 10.1158/1055-9965.EPI-09-0448 PMC2805163 20056649

[DMM032300C10] Grant W. B., Whiting S. J., Schwalfenberg G. K., Genuis S. J. and Kimball S. M. (2016). Estimated economic benefit of increasing 25-hydroxyvitamin D concentrations of Canadians to or above 100 nmol/L. *Dermatoendocrinology* 8, e1248324 10.1080/19381980.2016.1248324 PMC5129897 27942348

[DMM032300C11] Hong S. N., Kim J. H., Choe W. H., Lee S.-Y., Seol D. C., Moon H.-W., Hur M., Yun Y.-M., Sung I. K., Park H. S. et al. (2012). Circulating vitamin D and colorectal adenoma in asymptomatic average-risk individuals who underwent first screening colonoscopy: a case-control study. *Dig. Dis. Sci.* 57, 753-763. 10.1007/s10620-011-1926-1 21984438

[DMM032300C12] Hopkins M. H., Owen J., Ahearn T., Fedirko V., Flanders W. D., Jones D. P. and Bostick R. M. (2011). Effects of supplemental vitamin D and calcium on biomarkers of inflammation in colorectal adenoma patients: a randomized, controlled clinical trial. *Cancer Prev. Res.* 4, 1645-1654. 10.1158/1940-6207.CAPR-11-0105 PMC3188339 21724580

[DMM032300C13] Irving A. A., Yoshimi K., Hart M. L., Parker T., Clipson L., Ford M. R., Kuramoto T., Dove W. F. and Amos-Landgraf J. M. (2014). The utility of Apc-mutant rats in modeling human colon cancer. *Dis. Model. Mech.* 7, 1215-1225. 10.1242/dmm.016980 25288683 PMC4213726

[DMM032300C14] Irving A. A., Plum L. A., Blaser W. J., Ford M. R., Weng C., Clipson L., DeLuca H. F. and Dove W. F. (2015). Cholecalciferol or 25-hydroxycholecalciferol neither prevents nor treats adenomas in a rat model of familial colon cancer. *J. Nutr.* 145, 291-298. 10.3945/jn.114.204396 25644350 PMC4304025

[DMM032300C15] Kumagai T., O'Kelly J., Said J. W. and Koeffler H. P. (2003). Vitamin D2 analog 19-nor-1,25-dihydroxyvitamin D2: antitumor activity against leukemia, myeloma, and colon cancer cells. *J. Natl. Cancer Inst.* 95, 896-905. 10.1093/jnci/95.12.896 12813173

[DMM032300C16] Lehmann E. L. (1998). *Nonparametrics: Statistical Methods Based on Ranks*. Revised Fi Upper Saddle River, New Jersey: Prentice Hall.

[DMM032300C17] Liu Y., Chen L., Zhi C., Shen M., Sun W., Miao D. and Yuan X. (2016). 1,25(OH)2D3 deficiency induces colon inflammation via secretion of senescence-associated inflammatory cytokines. *PLoS ONE* 11, e0146426 10.1371/journal.pone.0146426 26790152 PMC4720393

[DMM032300C18] Pommergaard H.-C., Burcharth J., Rosenberg J. and Raskov H. (2016). Aspirin, calcitriol, and calcium do not prevent adenoma recurrence in a randomized controlled trial. *Gastroenterology* 150, 114-122.e4. 10.1053/j.gastro.2015.09.010 26404953

[DMM032300C19] Rebel H., der Spek C. D.-V., Salvatori D., van Leeuwen J. P. T. M., Robanus-Maandag E. C. and de Gruijl F. R. (2015). UV exposure inhibits intestinal tumor growth and progression to malignancy in intestine-specific Apc mutant mice kept on low vitamin D diet. *Int. J. Cancer* 136, 271-277. 10.1002/ijc.29002 24890436

[DMM032300C20] Takahashi R., Mizoue T., Otake T., Fukumoto J., Tajima O., Tabata S., Abe H., Ohnaka K. and Kono S. (2010). Circulating vitamin D and colorectal adenomas in Japanese men. *Cancer Sci.* 101, 1695-1700. 10.1111/j.1349-7006.2010.01575.x 20507319 PMC11158427

[DMM032300C21] Tangpricha V., Flanagan J. N., Whitlatch L. W., Tseng C. C., Chen T. C., Holt P. R., Lipkin M. S. and Holick M. F. (2001). 25-hydroxyvitamin D-1alpha-hydroxylase in normal and malignant colon tissue. *Lancet* 357, 1673-1674. 10.1016/S0140-6736(00)04831-5 11425375

[DMM032300C22] van der Rhee H., Coebergh J. W. and de Vries E. (2013). Is prevention of cancer by sun exposure more than just the effect of vitamin D? A systematic review of epidemiological studies. *Eur. J. Cancer* 49, 1422-1436. 10.1016/j.ejca.2012.11.001 23237739

[DMM032300C23] Vieth R. (2011). Why the minimum desirable serum 25-hydroxyvitamin D level should be 75 nmol/L (30 ng/ml). *Best Pract. Res. Clin. Endocrinol. Metab.* 25, 681-691. 10.1016/j.beem.2011.06.009 21872808

[DMM032300C24] Wang Y., Marling S. J., Zhu J. G., Severson K. S. and DeLuca H. F. (2012). Development of experimental autoimmune encephalomyelitis (EAE) in mice requires vitamin D and the vitamin D receptor. *Proc. Natl Acad. Sci. USA* 109, 8501-8504. 10.1073/pnas.1206054109 22592802 PMC3365177

[DMM032300C25] Washington M. K., Powell A. E., Sullivan R., Sundberg J. P., Wright N., Coffey R. J. and Dove W. F. (2013). Pathology of rodent models of intestinal cancer: progress report and recommendations. *Gastroenterology* 144, 705-717. 10.1053/j.gastro.2013.01.067 23415801 PMC3660997

[DMM032300C26] Wierzbicka J. M., Binek A., Ahrends T., Nowacka J. D., Szydłowska A., Turczyk Ł., Wąsiewicz T., Wierzbicki P. M., Sądej R., Tuckey R. C. et al. (2015). Differential antitumor effects of vitamin D analogues on colorectal carcinoma in culture. *Int. J. Oncol.* 47, 1084-1096. 10.3892/ijo.2015.3088 26260259 PMC4532196

[DMM032300C27] Wu K., Feskanich D., Fuchs C. S., Willett W. C., Hollis B. W. and Giovannucci E. L. (2007). A nested case-control study of plasma 25-hydroxyvitamin D concentrations and risk of colorectal cancer. *J. Natl. Cancer Inst.* 99, 1120-1129. 10.1093/jnci/djm038 17623801

[DMM032300C28] Yang L. and Toriola A. T. (2017). Leisure-time physical activity and circulating 25-hydroxyvitamin D levels in cancer survivors: a cross-sectional analysis using data from the US National Health and Nutrition Examination Survey. *BMJ Open* 7, e016064 10.1136/bmjopen-2017-016064 PMC5541594 28698340

[DMM032300C29] Yang B., Gross M. D., Fedirko V., McCullough M. L. and Bostick R. M. (2015). Effects of calcium supplementation on biomarkers of inflammation and oxidative stress in colorectal adenoma patients: a randomized controlled trial. *Cancer Prev. Res.* 8, 1069-1075. 10.1158/1940-6207.CAPR-15-0168 26304464

[DMM032300C30] Yi Pan S., Morrison Sai Yi Pan H., Morrison H. and Hishida A. (2011). Epidemiology of cancer of the small intestine. *World J. Gastrointest. Oncol.* 3, 33-42. 10.4251/wjgo.v3.i3.33 21461167 PMC3069308

[DMM032300C31] Zhang X., Keum N. N., Wu K., Smith-Warner S. A., Ogino S., Chan A. T., Fuchs C. S. and Giovannucci E. L. (2016). Calcium intake and colorectal cancer risk: results from the nurses’ health study and health professionals follow-up study. *Int. J. Cancer* 139, 2232-2242. 10.1002/ijc.30293 27466215 PMC5017917

