# Supplementary material for: Vitamin D deficiency in the ApcPirc/+ rat does not exacerbate colonic tumorigenesis, while low dietary calcium might be protective
Source: Dis Model Mech. 2018 Mar 1;11(3):dmm032300. doi: 10.1242/dmm.032300 (PMC5897728; doi:10.1242/dmm.032300)
Supplement: First Person interview [file dmm-11-032300-s1.pdf]

FIRST PERSON

First person – Amy Irving

First Person is a series of interviews with the first authors of a selection of papers published in Disease Models & Mechanisms, helping early-career researchers promote themselves alongside their papers. Amy Irving is first author on 'Vitamin D deficiency in the *Apc*$^{Pirc/+}$ rat does not exacerbate colonic tumorigenesis, while low dietary calcium might be protective', published in DMM. Amy is a postdoctoral research associate in the lab of Hector DeLuca at University of Wisconsin-Madison, Madison, USA, investigating the individual roles of vitamin D, calcium and ultraviolet light on the initiation, growth and progression of colonic tumors using highly controlled animal models, which provide the groundwork for translation to the clinic.

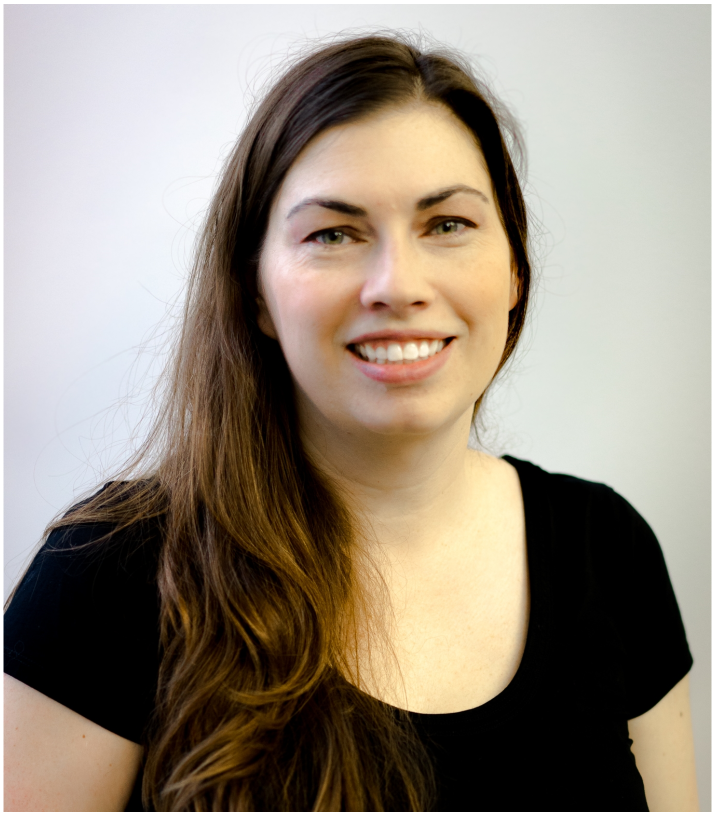
Amy Irving

How would you explain the main findings of your paper to non-scientific family and friends?

Colorectal cancer (CRC) is one of the leading causes of cancer globally. The disease generally occurs later in life and therefore has great potential for early intervention by altering environmental exposures. Reduced sunlight exposure is one factor that has been associated with increased colon cancer risk. Because sunlight exposure drives vitamin D production in the skin, early researchers believed that vitamin D had the ability to suppress CRC. However, our most recent research challenges the premise that vitamin D deficiency directly causes an increase in CRC. We have used a rat model of intestinal tumorigenesis, where all vitamin D is removed from their diet and their lighting is shielded to eliminate the wavelengths responsible for vitamin D production in skin. Despite this complete lack of vitamin D, we saw no difference in tumor incidence, development, growth, clinical pathology or susceptibility to colonic inflammation in deficient animals compared with those who were vitamin D sufficient. Similarly, clinical trials using vitamin D to supplement deficient patients in an effort to reduce colon cancer development have thus far shown little promise. Determining the true active players in colon cancer risk is crucial to impacting the disease, including a clear distinction between the effects of vitamin D and sunlight exposure itself.

What are the potential implications of these results for your field of research?

Although this finding is counter to popular opinion and years of productive research, we believe it will encourage the field as a whole to diversify the search into factors more probable than vitamin D as the causative risk driver within the location association paradigm. Harkening back to the original observation of the global distribution of CRC, one possible alternate is sunlight exposure itself. Vitamin D levels and sunlight exposure are inextricably linked in human populations, and studies in cell culture lack the systemic complexity to fully interrogate this question. Animal models thus fill an essential gap to aid our understanding of the system as a whole.

Amy Irving's contact details: University of Wisconsin-Madison, Department of Biochemistry, Madison, WI 53706.

E-mail: aairving@wisc.edu

What are the main advantages and drawbacks of the model system you have used as it relates to the disease you are investigating?

The Pirc rat model of colonic tumorigenesis has many advantages. The *Apc* gene mutated in the Pirc rat is also present in ~80% of human colon cancers, causing the development of multiple colonic tumors within a few months of age with nearly 100% incidence. Additionally, the mutation has been introduced into several different rat strains, allowing investigation of diversity in response to experimental conditions. And crucially, the reliable phenotype of the model allows us to carefully manipulate individual aspects of the animal's environment in a controlled manner, be it dietary vitamin D, calcium or ultraviolet light exposure. The main drawback of the Pirc rat model is that tumors rarely progress to invasive stages without additional treatments. However, this gives us the opportunity to explicitly examine how different environmental factors specifically influence early stages of disease.

What has surprised you the most while conducting your research?

What surprised us the most during the course of these experiments was our finding that low calcium seems to suppress colon tumor development. Other published studies have pointed at calcium being protective but we have shown the opposite. We have the unique opportunity to be able to modulate vitamin D and calcium

independently to investigate how they work together. Understanding the feedback mechanisms at play in this system is key to understanding which are drivers of disease and which are simply passengers.

"Understanding the feedback mechanisms at play in this system is key to understanding which are drivers of disease and which are simply passengers."

What changes do you think could improve the professional lives of early-career scientists?

I think a paradigm shift in the expectations of an early-career scientist is paramount to retaining the investment made in individuals following graduate school. Many scientists are launching their careers during a time when they are also caring for young families, often as part of a household where both parents are professionals. We need to create a path for scientists who wish to pursue faculty positions but delay or slow their scientific commitments when family needs are most demanding. A more supportive system to guide the transition from graduate studies to becoming productive, influential faculty is crucial in the development of a young scientist. As postdoctoral researchers, we often find ourselves without the same opportunities that allowed us to bloom in graduate school, such as direction by a faculty committee or opportunities to present our research, unless we are actively pursuing early faculty positions.

What's next for you?

My interests are deeply rooted in environmental toxicology, from the standpoint of how environmental factors perturb disease progression and overall survival. Colon cancer and multiple sclerosis, two highly prevalent diseases, have each been associated with vitamin D levels, but neither have clearly demonstrated improvement with vitamin D supplementation. It is my goal to continue to delineate and clarify any role of vitamin D or related environmental factors such as sunlight, and their impact on human health and disease.

Reference

Irving, A. A., Duchow, E. G., Plum, L. A. and DeLuca, H. F. (2018). Vitamin D deficiency in the *Apc*$^{Pirc/+}$ rat does not exacerbate colonic tumorigenesis, while low dietary calcium might be protective. *Dis. Model. Mech.* **11:** dmm032300.
